# Moral distress among residents in neurology: a pilot study

**DOI:** 10.1186/s42466-021-00104-5

**Published:** 2021-02-01

**Authors:** Hanna Hildesheim, Annette Rogge, Christoph Borzikowsky, Victoria Dorothea Witt, Eva Schäffer, Daniela Berg

**Affiliations:** 1 Department of Neurology, Kiel University, University-Hospital-Schleswig-Holstein, Arnold-Heller-Str. 3, 24105 Kiel, Germany; 2 Institute for Experimental Medicine, Medical Ethics, Kiel University, University-Hospital-Schleswig-Holstein, Kiel, Germany; 3 Institute of Medical Informatics and Statistics, Kiel University, University-Hospital-Schleswig-Holstein, Kiel, Germany; 4 Department of Neurology, Segeberger Kliniken, Bad Segeberg, Germany

**Keywords:** Moral distress, Working conditions, Residents in neurology

## Abstract

**Background:**

Medical progress, economization of healthcare systems, and scarcity of resources raise fundamental ethical issues. Physicians are exposed to increasing moral conflict situations, which may cause Moral Distress (MD). MD occurs when someone thinks he or she might know the morally correct action but cannot act upon this knowledge because of in- or external constraints. Correlations of MD among residents to job changes and burn-out have been shown previously. There are, however, hardly any quantitative studies about MD among physicians in Germany. The aim of this study was to investigate the frequency of occurrence, the level of disturbance, and reasons for MD among neurological residents in German hospitals.

**Methods:**

1st qualitative phase: Open interviews on workload and ethical conflicts in everyday clinical practice were conducted with five neurological residents. Ethical principles of medical action and potential constraints that could cause MD were identified and a questionnaire designed. 2nd quantitative phase: A preliminary questionnaire was tested and evaluated by five further neurological residents. The final questionnaire consisted of 12 items and was conducted online and anonymously via e-mail or on-site as part of an unrelated resident training event at 56 sites.

**Results:**

One hundred seven neurological residents from 56 university/acute care and rehabilitation hospitals throughout Germany were examined (response rate of those requesting the questionnaire: 75.1%). 96.3% of the participants had experienced MD weekly (3.86, SD 1.02), because they were unable to invest the necessary time in a patient or relative consultation. Errors in medical care, which could not be communicated adequately with patients or relatives, were rated as most distressing. The most common reasons for MD were the growing numbers of patients, expectations of patient relatives, fears of legal consequences, incentives of the DRG-system, and the increasing bureaucratization requirement. 43.0% of participants mentioned they considered leaving the field of inpatient-care. 65.4% stated they would like more support in conflict situations.

**Conclusion:**

MD plays an important role for neurological residents in German hospitals and has an impact on participants’ consideration of changing the workplace. Important aspects are rationing (time/beds) and incentives for overdiagnosis as well as lack of internal communication culture and mentoring.

**Supplementary Information:**

The online version contains supplementary material available at 10.1186/s42466-021-00104-5.

## Background

Moral distress (MD) is a psychological construct developed in 1984 by the US-American philosopher and nursing-ethicist Andrew Jameton. According to him, a person experiences MD when *“one knows the right thing to do, but institutional constraints make it nearly impossible to pursue the right course of action”* ([1], p.6). Other authors extended the concept for the medical field and distinguished between:

- external constraints (e.g., inadequate staffing and increase turnover, lack of administrative support, hierarchies within health-care system [2])
- or internal constraints (e.g., perceived powerlessness, lack of knowledge of alternative treatment plans, inability to identify ethical issues [2])

Thus, originally MD differs from the definition of the classical philosophical dilemma in which someone does not know how to act because both given opportunities would infringe an ethical principle. Recently, authors broadened the original definition and described MD as a “*psychological response to morally challenging situations such as those of moral constraint or moral conflict, or both.”* ([3], p. 97).

MD research can generate knowledge on two levels: On the one hand, it reveals new aspects of mental health and job satisfaction of health care system workers: Physicians who experienced MD in their daily clinical routine suffered significantly more often from symptoms of depression [4] and burn-out [5]. In several studies, physicians who experienced MD indicated that they had already changed jobs or were currently thinking about changing the workplace [2, 6]. On the other hand, the MD concept highlights the constraints and conflict situations which lead to MD. It can, therefore, provide insights into problem areas and deficits of a health care system and subsequently into potential of improvement. While the main reasons of MD in Iran were lack of medication and medical equipment [7], physicians working in intensive care units in the UK suffered mostly from futile medical treatment [6].

There is little research on MD explicitly among residents. A study from Canada shows that residents encountered MD when they observed patient consultations or behaviour they perceived as too short or as unprofessional or not appropriate [8]. Other studies coincidence with the already described results: residents experienced significant MD from witnessing specific treatments in the end-of-life-care they perceived as futile [9] and their level of MD correlated positively with burn-out and the intention to leave work place [10].

These examples demonstrate how the concept of MD differs from occupational stress models such as the effort-reward-imbalance model [11] or the job-demand-control model [12]. Moreover, they indicate how various reasons for MD can be in different countries and cultures. A major shortcoming of many MD scores is the need for a cultural adaption and the need for a differentiated consideration of different medical professions, respectively other health care providers.

It was shown from occupational stress models that 63.9% of the neurological residents in Germany suffer job associated strains [13]. It has also been pointed out that young physicians in Germany criticise the documentation requirements, their specialist training, workload, shortage of staff, and the influence of the economy as most important aspects that should be improved in their working environment [14, 15]. Moreover, it was shown for German residents working in intensive care units that perception of futile treatment leads to burn-out and intention to leave the job [16]. But so far, there are hardly any quantitative studies on MD in Germany.

In particular there are no validated MD measurement instruments for residents, physicians working in neurology or the German health care system. Two German studies about MD among medical students indicated that MD might also plays an important role in Germany [17, 18]. In order to respond to an identified research gap in Germany [19] this pilot study was designed to gain first insights on relevance and impact of MD in Germany. For three main reasons it was therefore required to design a new MD measurement instrument:

- So far there is no preliminary information about MD among the investigated focus group for deriving some hypotheses. We therefore decided to investigate the conditions that may lead to MD. We decided that construct validation should be tested in a further study, if it would become apparent, that MD plays a relevant role for the participants.
- As mentioned above it was assumed that MD differs not only between culture, health care system and specialization, but also among hierarchies.
- A main aspect of this study was to investigate and quantify constraints leading to MD, in order to draw attention to reasons why physicians are obliged to infringe their ethical principles and derive recommendations for the improvement of working conditions or specialist training.

## Methods

### Study design

**Fig. 1:**
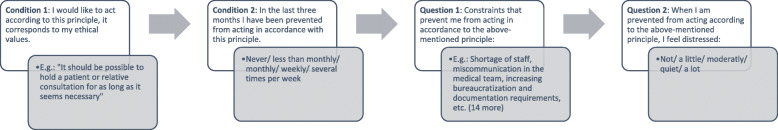
Structure of the questionnaire demonstrated by exemplary use of item 1

Conditions for the experience of MD are that somebody considers an action as ethically correct (Condition 1) and that he or she is prevented from acting in accordance to that (Condition 2). For the development of the questionnaire of this study only those participants who experienced these MD-conditions were asked to answer a set of questions (see Fig. 1) about subjective reasons for MD and the level of disturbance.

Since the original questionnaire is in German, the English translation of the items should be understood analogously and not suitable for use. The study was approved by the local ethics committee of the University of Kiel (application number: D 581/18) and participants’ written informed consent was obtained.

### Instrument development

1st qualitative phase: As a preliminary study five residents from three different neurological departments in Germany were asked in open interviews about ethical conflicts and general workload in everyday clinical practice.

The ethical conflicts mentioned in the preliminary study and the MD conflict situations of four already existing MD-measuring instruments [20–24] were split into (1) the infringed ethical principle and (2) the MD causing constraints. They were summed up thematically and checked regarding their compatibility with the German health care system. Thus, 14 new items and a list of 13 constraints were compiled (see Table 2). The five-point Likert scale on level of disturbance was labelled according to Rohrmann [25] and another five-point Likert scale to frequency, relating on the frequency of occurrence in the last 3 months, was labelled according to Kleinknecht et al. ([23]; see Table 2). The first version of the questionnaire was discussed in an interdisciplinary working group (consisting of the first, second, and last author of this paper as well as an specialist for medical ethics) and as a result of literature research and discussion three further constraints were added (fear of legal consequences, wishes, and expectations of patient relatives, guidelines of the administration). During open interviews of the 1st qualitative phase “lack of time” was a frequently mentioned constraint. As it was assumed that “lack of time” itself is the result of shortcomings such as, e.g., poor organization and understaffing and as it often serves as a mediator between constraint and MD-situation, it was deliberately refrained from using it as an answer option in this questionnaire.

2nd quantitative test phase: The questionnaire was designed as a web-survey using survey-software Evasys (Version 7.0, Electric Paper Ltd.; 2016) and tested by five other residents from three further neurological departments, a psychologist with expertise in medical ethics as well as a psychologist with specialization in statistics. An evaluation sheet was attached to the study (see additional file 1).

After revision of the questionnaire and further discussion in the working group, two items on the topic of respectful interaction with each other were deleted, as these were estimated as too unspecific. As a further constraint “miscoordination with other medical specializations” was added. It was decided to additionally paraphrase the words “ethical” and “moral”, since the preliminary study had shown that these words were interpreted differently. The final version abstained from a definition of MD at the beginning, to allow participation without exerting influence by the basic concept or the chosen definition. The questionnaire included questions about socio-demographic data, request for support, and intention to leave the field of inpatient-care. If a participant mentioned that he considered to leave the field of inpatient-care, he could rate on a five-point Likert scale (from “1 = not” to “5 = a lot”) as labelled by Rohrmann how this intention was related to the conflict situations mentioned in the questionnaire [25]. The original questionnaire can be referred on the authors website ([26]). It took about 15 min to complete the questionnaire (Fig. 1).

### Procedure

Neurological departments of university, acute care, and rehabilitation hospitals were contacted to recruit volunteers. If the request was accepted by the chief physician, the department’s residents were contacted by e-mail or the survey was conducted on-site alongside an unrelated resident training event. The survey period was from 21.02.2019 to 13.06.2019.

### Statistical analysis

Statistical analysis was conducted with SPSS for Windows (Version 21, IBM, 2012). A descriptive analysis of the items with absolute and relative frequencies of the response categories was carried out. For group comparisons, the Mann-Whitney U-test and the Kruskal-Wallis test were used. Correlations between variables were analysed with correlation coefficients according to Pearson. A *p*-value below 0.05 was considered as statistically significant.

## Results

Thirty-six university hospitals and 137 acute care and rehabilitation hospitals throughout Germany were contacted. Fifty-six chief physicians agreed that their residents could participate in the study. In eight of those 56 participating hospitals on-site surveys with residents were conducted alongside their unrelated internal training. Forty-eight chief physicians passed on the request to participate to their neurological residents, of whom 98 residents agreed to participate via e-mail. In total, 145 residents requested access to the questionnaire, of which 109 recorded and returned their answers (response rate: 75.17%). Finally, 107 data sets were evaluable (approval of the data protection declaration, fulfilment of the inclusion criteria, etc.). Items were not evaluated in case the conditions for MD-experience (ethical principle and occurrence of infringement) were not specified.

The participants were on average in their fourth year of medical practice (3.66; SD 2.04) and 31 years old (30.81; SD 3.32). 61.7% of the participants were female, 35.5% male, and 2.8% did not specify (Table 1).

**Table 1:** Socio-demographic data

Characteristics	Descripition	n (%), mean (SD)
Gender	Male	38 (35.5)
	Female	66 (61.7)
	Not specified	3 (2.8)
Age		30.81 (SD 3.32)
Year of specialist training		3.66 (SD 2.04)
Years already worked as a physician		3.93 (SD 2.47)
Working hours per week		50.17 (SD 9.06)
Type of hospital	Acute care clinic	48 (44.9)
	University Hospital	48 (44.9)
	Rehabilitation clinic	7 (6.5)
	Other	3 (2.8)
	Missing	1 (0.9)
Owner of the clinic	Public	57 (53.3)
	Charitable	10 (9.3)
	Private	26 (24.3)
	I do not know	12 (11.2)
	Missing	2 (1.9)

On average participants experienced seven out of twelve MD-conflict-situations mentioned in the questionnaire in the last 3 months [7.03 (SD 2.35)]. The occurrence of MD varied according to the conflict situation. 96.3% of participants and thus most frequently [on average “weekly” (3.86; SD 1.02) stated that they had experienced MD because they were unable to hold a patient or relative consultation as long as necessary (Item 1). The main barriers for this frequent reason of MD were “growing numbers of patients” (74.0%) and the “increasing bureaucratization and documentation requirements” (60.6%). The experience of MD related to diagnostics or therapy without the patient’s consent occurred in about 1/3 of the participants (Item 5: 35.5%; Item 3: 33.6%).

The average level of disturbance of all participants was 3.36 (SD 0.64), which means “moderately” distressing according to the verbal Likert scale. Errors in medical care that could not be communicated with patients or relatives were perceived as most distressing [3.74 (“quiet”); SD 0.94] (Item 7). “Fear of legal consequences” (33%) and “lack of provider continuity (changing the ward/ shift work)” (37.4%) were mentioned as main reasons for this (Table 2).

**Table 2:** All items, sorted by occurrence (descending); frequency scale (five-point Likert scale): 1 = nie (“never”), 2 = seltener als monatlich (“less then monthly”), 3 = monatlich (“monthly”), 4 = wöchentlich (“weekly”), 5 = mehrmals wöchentlich (“several times per week”); level of disturbance (five-point Likert scale): 1 = nicht (“not”), 0 = wenig (“a little”), 3 = mittelmäßig (“moderatly”), 4 = ziemlich (“quiet”), 5 = sehr (“a lot”)

Ethical principle	Occurence in three last three months N (%)	Frequency of occurrence Mean (SD)	Level of disturbance Mean (SD)	Main constraints N (%)
It should be possible to hold a patient or relative consultation for as long as it seems necessary. (Item 1)	103 (96.3)	3.86 (1.02)	3.29 (0.75)	1. Growing patient population *n* = 77 (74) 2. Increasing bureaucratization and documentation requirements *n* = 63 (60.6)
A patient should be able to stay on the ward for as long as is necessary to ensure the best possible treatment of the current complaints or to establish a clear perspective for the further procedure. (Item 9)	80 (74.8)	2.8 (1.24)	3.5 (0.97)	1. Lack of resources *n* = 53 (63.9) 2. Incentives of the DRG- system *n* = 47 (56.6)
Only those diagnostics should be performed that are essential for the diagnosis, understanding of the symptoms or therapy monitoring. (Item 2)	77 (72)	2.99 (1.21)	2.76 (0.85)	1. Fear of legal consequences *n* = 35 (44.9) 2. Incentives of the DRG- system *n* = 27 (34.6)
Misjudgments, misunderstandings and circumstances that have led to errors in medical care should be communicated honestly with patients and their relatives. (Item 7)	74 (69.2)	2 (0.75)	3.74 (0.94)	1. Fear of legal consequences *n* = 33 (44) 2. Lack of provider continuity *n* = 28 (37.3)
A patient who is not capable of giving consent should be treated according to his or her presumed will in cooperation with relatives, a Patient Decree and/or legal carer. (Item 6)	64 (59.8)	1.87 (0.87)	3.68 (0.9)	1. Wishes and expectations of patient relatives *N* = 41 (64.1) 2. Fear of legal consequences *n* = 20 (31.3)
Misjudgments, misunderstandings and circumstances that have led to errors in medical care should be communicated honestly within the team. (Item 8)	61 (57)	1.89 (0.94)	3.68 (0.89)	1. Lack of support of supervisors *n* = 28 (43.8) 2. Miscommunication in the medical team *n* = 27 (42.2)
Only those therapies or therapeutic attempts should be carried out for which a clearly formulated therapeutic objective appears to be achievable. (Item 4)	61 (57)	2.22 (1.06)	3.23 (0.84)	1. Wishes and expectations of patient relatives *n* = 32 (51.6) 2. Fear of legal consequences *n* = 25 (40.3)
Patients with private and statutory health insurance should be offered the same quality of medical care. (Item 12)	57 (53.3)	2.2 (1.32)	3.33 (1.13)	1. Incentives of the DRG- system *n* = 34 (57.6) 2. Guidelines of the administration *n* = 23 (39)
If it can be assumed that the therapeutic objective intended by the patient (e.g. life without invasive ventilation, return to home) can no longer be achieved, palliative therapy concepts should also be discussed. (Item 11)	54 (50.5)	1.8 (0.88)	3.7 (1.01)	1. Wishes and expectations of patient relatives *n* = 38 (67.9) 2. Fear of legal consequences *n* = 21 (37.5)
A patient should only be restrained or medically sedated as a last resort and for as short as possible in case of acute danger to oneself or others. (Item 10)	47 (43.9)	1.77 (0.99)	3.57 (0.89)	1. Shortage of staff *n* = 39 (83) 2. Growing patient population *n* = 20 (42.6)
In the case of a patient who is capable of giving consent just therapies should be carried out to which he or she has informed agreed. (Item 5)	38 (35.5)	1.73 (1.11)	3.14 (0.96)	1. Growing patient population *n* = 15 (38.5) 2. Increasing bureaucratization and documentation requirements *n* = 12 (30.8)
In the case of a patient who is capable of giving consent just diagnostics should be performed out to which he or she has informed agreed. (Item 3)	36 (33.6)	1.63 (1.00)	2.92 (1.09)	1. Growing patient population *n* = 13 (35.1) 2. Lack of provider continuity *n* = 12 (32.4)

No significant difference in the perception or level of disturbance in MD between men and women was detected. Age or year of specialist training and type or owner of the hospital did not correlate significantly with the average frequency of occurrence or average level of disturbance. The values of frequency and level of disturbance correlated significantly in five items with each other. When testing a partial correlation analysis excluding the factor “working hours per week”, just three items remained significant (Item 1: *r = .22; p* = .027; Item 8: *r* = 0.312; *p* = .018; Item 9: *r* = 0.402; *p* < .000).

65.4% of the participants stated that they would like to receive more support in the mentioned conflict situations. Of these, 52.9% asked for more support from their supervisors. 43.0% of the participants said they were considering leaving the field of inpatient-care. Comparing this consideration in relation to the conflict situations mentioned in the questionnaire the grading of the answer was “moderately” (3.15; SD 1.03).

### Intentions to leave job

**Fig. 2:**
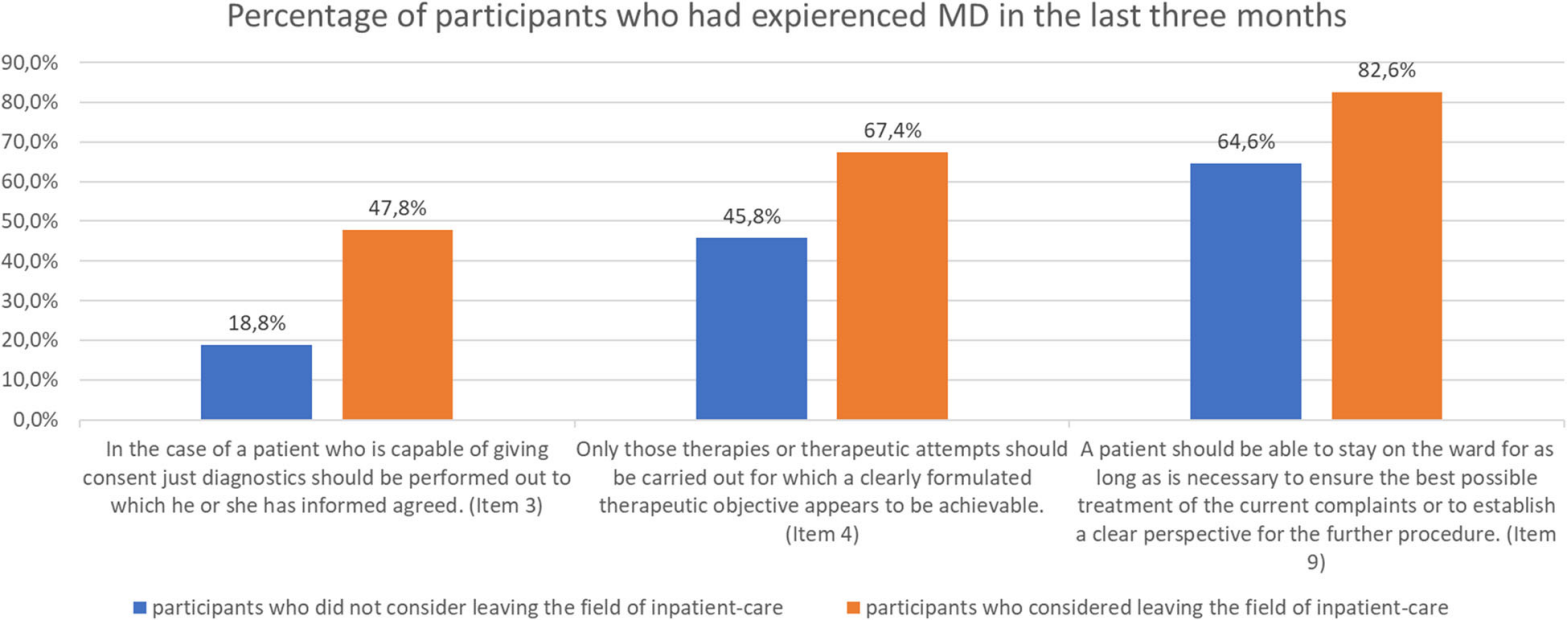
Significant differences between participants who considered leaving the field of inpatient-care and those who did not; Item 3: *p* = .003; Item 4: *p* = .036; Item 9: *p* = .049

Residents who indicated that they were considering leaving the field of inpatient-care were significantly older [“leavers”: 31.47 (SD 03.69) than “remainers”: 29.96 (SD 2.62); *p* = .034] and had been working significantly longer as physicians than those who did not consider [“leavers”: 4.74 (SD 2.95) to “remainers”: 3.28 (SD 1.85); *p* = .011]. They experienced three MD-situations significantly more frequently than those who did not consider leaving the field of inpatient-care (see Fig. 2). They perceived significantly more distress in situations in which a patient had been restrained or medically sedated before all other options had been tried or longer than absolutely necessary [Item 10, “leavers”: 3.96 (SD 0.93) to “remainers”: 3.17 (SD 0.17); *p* = .004]. Participants who considered leaving the field of inpatient-care did not perceive significantly higher average levels of disturbance of MD than participants who did not consider. However, on average participants who considered leaving the field of inpatient-care perceived significantly more frequently MD than the participants who did not consider to leave [average frequency: “leavers”: 2.38 (SD 0.5) to “remainers”: 2.13 (SD 0.5); *p* = .002]. The average frequency of the perception of MD correlated significantly with the level of agreement to considerations of leaving the field of inpatient-care due to conflict situations mentioned in the questionnaire (*r* = 0.462; *p* = .001).

### Constraints

Taking a closer look at the constraints of MD, the most common reason for MD over all items was the “growing numbers of patients “, respectively “shortage of staff”. Furthermore, frequent answers were “wishes and expectations of patient relatives”, “fear of legal consequences”, “incentives of the DRG-system”, and “increasing bureaucratization and documentation requirements”. A lack of qualification was rarely indicated. The specific constraints for each MD conflict situation are shown in Table 2.

## Discussion

Moral distress as a psychological response to challenging moral situations has a relevant impact on professionals in the healthcare system.

This study shows that neurological residents in Germany regularly suffer from MD in their daily work life. On average the participants had experienced seven of the twelve MD conflict situations in the last 3 months and 65.4% of the participants stated that they would like to receive more support in the mentioned conflict situations.

Comparable to studies from other countries on MD, the present study did not show any significant difference in frequency of occurrence or level of disturbance between women and men [4, 7]. Similarly, there was no significant correlation between the frequency of occurrence or level of disturbance with increasing age or year of specialist training. This aspect is supported by some comparable previous studies [27–29], yet not by all [22, 30]. Similar to comparable MD-studies the value of frequency and level of disturbance correlated only in a few items significantly in this study [31]. Thus, frequent occurrence did not lead to a higher or lower strain of MD in general.

The growing number of patients and increasing bureaucratization and documentation requirements in a situation of shortage of staff are central aspects for the genesis of MD according to the participants of this study. These findings largely coincide with the main requests for improvement in working conditions and specialist training of previous studies from Germany [14, 15] and the results of a recent study of Epstein et al. from the US about the main root causes of MD for physicians [32]. The study of Biesalski et al. highlighted the wish of neurological residents in Germany for more mentoring and a better feedback culture in their specialist training [14]. Also, in this present study 64.0% of the participants would like to receive more support in the painted MD-conflict situations. Raspe et al. focussed on psycho-social working strains of young physicians in Germany and their harmful consequences for physicians and patients [15]. Also, this present study shows that the working strain and discontent of neurological residents might be huge: nearly half of the participants indicated they were considering leaving the field of inpatient care.

This study also reveals “wishes and expectations of the patient’s relatives” as one of the main constraints of MD, since these were the main reasons for continuing life-supporting treatment (Item 11) and futile treatment in general (Item 4). It can be assumed that these expectations often arise from a lack of information about what is medically possible and reasonable but also what is in line with the legal framework. Since, according to the findings of this study a patient or relative consultation could not be held with the necessary frequency, length, and exhaustiveness, a vicious circle is revealed.

A “lack of qualification” was rarely indicated as a reason for MD. Thus, it can be assumed, that neurological residents in Germany feel adequately trained or supported regarding their medical expertise. By contrast, the “fear of legal consequences” was an often-mentioned constraint for MD in this study. Considering that only a very low percentage of physicians in Germany are legally sentenced, there seems to be a lack of information on individual legal risks for physicians.

Our findings about relatives’ expectations and fear of legal consequences highlight the frequent dilemma of complex end-of-life-decisions with increasing possibilities of intensive care especially in neurology with a high number of patients with disturbances of consciousness and rarely certain prognosis [33]. It was shown that in the last 15 years treatment limitations (withholding or withdrawing life-sustaining treatment or active shortening of the dying process) occurred significantly more frequently, whereas death without any limitations in life-prolonging therapies occurred significantly less frequently [34]. To reduce MD this decision processes require time, legal, and communicative expertise. We, therefore, recommend on the one hand an appropriate focus on these aspects in medical training and on the other hand a cultural discourse about dying and death in hospital and the importance of a Patient Drecree.

According to a study by the auditing company PricewatherhouseCoopers (PwC) of 2012, one in four physicians in Germany quits work as a physician early in the career and changes business completely (e.g., to administration or pharmacy). In addition to a peak of graduates who do not even begin to work as physicians, there is a second wave of emigration 8 to 12 years after entering the profession [35]. The results of the present study reinforce this: Neurological residents who considered leaving the field of inpatient-care were older and had worked longer as physicians. The study could not show that considerations of leaving inpatient-care correlated with a higher average level of disturbance as previous studies had shown [22, 36]. However, in the present study residents who considered leaving the field of inpatient-care experienced the mentioned conflict situations on average significantly more frequently. Hence it may be assumed that this identified at least one reason for migration of physicians. As a limitation, it should be noted that the questionnaire did not include distinctions whether participants considered changing in outpatient-care or were seeking a complete change of profession.

We designed a questionnaire that allows important insights into working conditions of neurological residents. It would be interesting to examine specialists and senior physicians separately to show the different demands in ethically challenging situations and investigate MD in Germany more in detail.

### Limitations

It is important to note that on first instance the chief physicians were asked for permission to seek participation, and only at second instance, the actual respondents participated voluntarily. Therefore, a certain selection bias must be assumed. Firstly, there is a possibility, with the chief physicians as “guardians” to participation, that study results reflect departments that were more open-minded for ethical topics. Previous studies have shown that a poor ethical climate correlates with higher levels of disturbance of MD [32]. Secondly, the respondents may already have been interested or have shown increased sensitivity to the topic before.

Another limitation that might account to additional selection bias is the small case number with participants mainly advanced in their residency.

This is an explorative study to analyse the significance of the construct MD among neurological residents in Germany. It is important to mention that this study investigated precisely the conditions leading to MD. The latent construct MD we discovered in our study must be proven in a further validation study. This further study also needs to provide information about reliability and robustness.

## Conclusions

MD plays an important role for neurological residents in Germany and has an impact on considerations to change the workplace for the participants.

Based on the present survey it has been possible to identify some aspects and reasons for MD, which seem to be of particular relevance and should be discussed at the micro- (team), meso- (hospital), and macro-level (politics):

- The main reason for MD is the increasing workload (shortage of staff, growing numbers of patients, increasing bureaucratization and documentation requirements), which comparable to previous studies highlights the importance of improving these working-conditions.
- Other important aspects which jeopardize medical empowerment are “wishes and expectations of patient relatives” and “fear of legal consequences”. Sufficient time for counselling of relatives and teaching on medico-legal aspects should be implemented in the training of residents.
- Situations identified as most stressful are those that arise from a lack of internal communication culture. An intentional establishment of communication structures and training might reduce MD.
- The most frequently mentioned MD-situations are rationing (consultation time, beds) and overdiagnosis. Our data shows that allocation at micro- and meso-level interfere with the resident’s values of patient-centred care. Simultaneously existing incentives for futile therapy and diagnostics aggravate a field of tension. This whole field therefore requires a political-social discourse.

## Supplementary Information

**Additional file 1.** The evaluation sheet used in quantitative test phase.

## Data Availability

The datasets used and analysed during the current study are available from the corresponding author on reasonable request.

## Abbreviations

MD: Moral Distress
DRG: Diagnosis related groups
