# Supplementary material for: Moral distress among residents in neurology: a pilot study
Source: Neurol Res Pract. 2021 Feb 1;3:6. doi: 10.1186/s42466-021-00104-5 (PMC7849144; doi:10.1186/s42466-021-00104-5)
Supplement: Supplementary file 1 — Additional file 1. The evaluation sheet used in quantitative test phase. [file 42466_2021_104_MOESM1_ESM.pdf]

Additional file 1: The evaluation sheet used in quantitative test phase

Evaluation sheet / Questions
How long did it take you to complete the questionnaire?
If someone would have given you the link to this online- questionnaire, would you have participated in the study?
Would you have finished the questionnaire completely?
Would you have preferred to select just one constraint per item instead of three?
Other comments, criticism and suggestions for improvement:
